# The relation between significant stenosis or dilatation at the repair site and outcome in a contemporary cohort of patients with repaired aortic coarctation as assessed using Cardiovascular Magnetic Resonance

**DOI:** 10.1186/1532-429X-14-S1-P116

**Published:** 2012-02-01

**Authors:** Sylvia S Chen, Konstantinos Dimopoulos, Rafael Alonso-Gonzalez, Emmanouil Liodakis, Elvis Teijeira-Fernandez, Maria Alvarez-Barredo, Gerhard-Paul Diller, Daryl Shore, Anselm Uebing, Lorna Swan, Michael Gatzoulis, Raad H Mohiaddin

**Affiliations:** 1 CMR Unit, Royal Brompton Hospital, London, UK; 2 Imperial College, London, UK

## Summary

Complications of restenosis or dilatation at the site of repair may occur late after initial repair of coarctation of the aorta (CoA). Cardiovascular magnetic resonance (CMR) is becoming the choice of imaging of patients after CoA. Using CMR, we studied the relation between these complications and outcome inn patients with previous CoA repair, and found that significant stenosis or dilatation at the repair site is a predictor of adverse outcome.

## Background

Cardiovascular magnetic resonance (CMR) is becoming the method of choice for assessing repaired CoA patients, especially for identifying important long-term sequelae of restenosis or dilatation at the repair site. We examined the prevalence of restenosis and dilatation at the site of aortic coarctation (CoA) repair and their relation to long-term outcome in patients with repaired CoA.

## Methods

Data and imaging for adult CoA patients followed in our institution were analysed. CMR images of the aorta were studied, and the diameter of the aorta at the repair site was measured on CMR and its ratio to the aortic diameter at the diaphragm (repair site-diaphragm ratio, RDR) calculated. Significant restenosis was defined as RDR≤50% and dilatation as RDR>150%. The relation of the CMR findings to the combined endpoint of death, stroke, surgery, intervention or escalation of antihypertensive therapy was assessed using Cox analysis.

## Results

A total of 247 patients (33.0±12.8 years, 59.5% male) were studied. Significant restenosis was present in 9.3% of patients, while 12.6% had significant dilatation. A discrete aneurysm at the repair site was observed in 8.5%. Restenosis was more likely in end-end anastomosis repairs and dilatation in patch repairs. Patients with restenosis were at higher risk of the combined endpoint (HR 4.26, 95%CI:1.83-9.96, p=0.001), as were patients with dilatation (HR 2.41, 95%CI:1.08-5.39, p=0.03). Hypertension was a significant predictor of outcome, even after exclusion of the “escalation of antihypertensive treatment” component (HR 3.95, 95%CI:1.19-13.05, p=0.02).

## Conclusions

Restenosis or dilatation at the CoA repair site as assessed by CMR is a predictor of adverse outcome.

## Funding

This study did not have any supporting funding.

